# Irradiation dependent inflammatory response may enhance satellite cell engraftment

**DOI:** 10.1038/s41598-020-68098-9

**Published:** 2020-07-06

**Authors:** Bruno Doreste, Silvia Torelli, Jennifer Morgan

**Affiliations:** 1 Dubowitz Neuromuscular Centre, Molecular Neurosciences Section, Developmental Neurosciences Research and Teaching Department, University College London Great Ormond Street Institute of Child Health, 30 Guilford Street, London, WC1N1EH UK; 2 NIHR Great Ormond Street Hospital Biomedical Research Centre, 30 Guilford Street, London, WC1N 1EH UK

**Keywords:** Cell biology, Stem cells

## Abstract

Skeletal muscle stem (satellite) cells transplanted into host mouse muscles contribute to muscle regeneration. Irradiation of host muscle enhances donor stem cell engraftment by promoting the proliferation of transplanted donor cells. We hypothesised that, similar to other systems, cells damaged by radiation might be effecting this donor cell proliferation. But we found no difference in the percentage of dying (TUNEL+) cells in immunodeficient dystrophic mouse muscles at the times after the irradiation dose that enhances donor cell engraftment. Similarly, irradiation did not significantly increase the number of TUNEL+ cells in non-dystrophic immunodeficient mouse muscles and it only slightly enhanced donor satellite cell engraftment in this mouse strain, suggesting either that the effector cells are present in greater numbers within dystrophic muscle, or that an innate immune response is required for effective donor cell engraftment. Donor cell engraftment within non-irradiated dystrophic host mouse muscles was not enhanced if they were transplanted with either satellite cells, or myofibres, derived from irradiated dystrophic mouse muscle. But a mixture of cells from irradiated muscle transplanted with donor satellite cells promoted donor cell engraftment in a few instances, suggesting that a rare, yet to be identified, cell type within irradiated dystrophic muscle enhances the donor stem cell-mediated regeneration. The mechanism by which cells within irradiated host muscle promote donor cell engraftment remains elusive.

## Introduction

Stem cell transplantation is a possible means of treating inherited neuromuscular diseases such as Duchenne muscular dystrophy (DMD), but environmental factors within skeletal muscle affect the engraftment efficiency of the transplanted cells. We have shown previously that high dose irradiation creates an environment that is favourable to donor stem cell proliferation and engraftment^1–3^. While high dose irradiation cannot be used in a clinical context, defining the underlying molecular mechanism(s) could suggest alternative clinically-safe approaches to modulate the host muscle environment and boost donor cell engraftment.

In our previous work, we have suggested that irradiation of host muscle facilitates donor cell engraftment by incapacitating or partially depleting the endogenous stem cell population^3^. But we also observed that irradiation leads to the extensive proliferation of donor stem cells and their progeny (myoblasts)^1,2^. This suggests that, as well as depletion of the host satellite cell niche, factors that promote or prolong the proliferation, and possibly delay the differentiation, of satellite cell progeny are being produced in host skeletal muscle as a response to radiation.

In non-muscle systems, is known that irradiated fibroblasts and melanoma cells act on neighbouring non-irradiated cells by the “bystander effect”, inducing chromosomal aberrations^4^, apoptosis^5^, or altered gene expression^6^ within them. Damaged cells in Drosophila and mouse models have been shown to induce the proliferation of nearby cells, promoting wound healing and tissue regeneration^7–9^. We therefore hypothesized that irradiation might enhance donor stem cell proliferation by damaging host cells and thus inducing the production of proliferative factors.

To investigate this, we performed RNA sequencing on irradiated and non-irradiated mdx nude mouse muscles and quantified TUNEL+ cells within non-irradiated and non-irradiated muscles of dystrophic and non-dystrophic immunodeficient mice. We also performed co-transplant experiments to determine whether cells from irradiated dystrophic mouse muscles could enhance engraftment of satellite cells derived from normal donor mice within non-irradiated dystrophic host mouse muscles. Our findings suggest that activation of the innate immune system within irradiated muscle is responsible for enhancing donor satellite cell engraftment. We further suggest that non-muscle cells within irradiated muscle are mediating this effect.

## Results

### Radiation of *mdx*^*nu/nu*^ mouse muscle elicits an innate immune response

**Figure 1:**
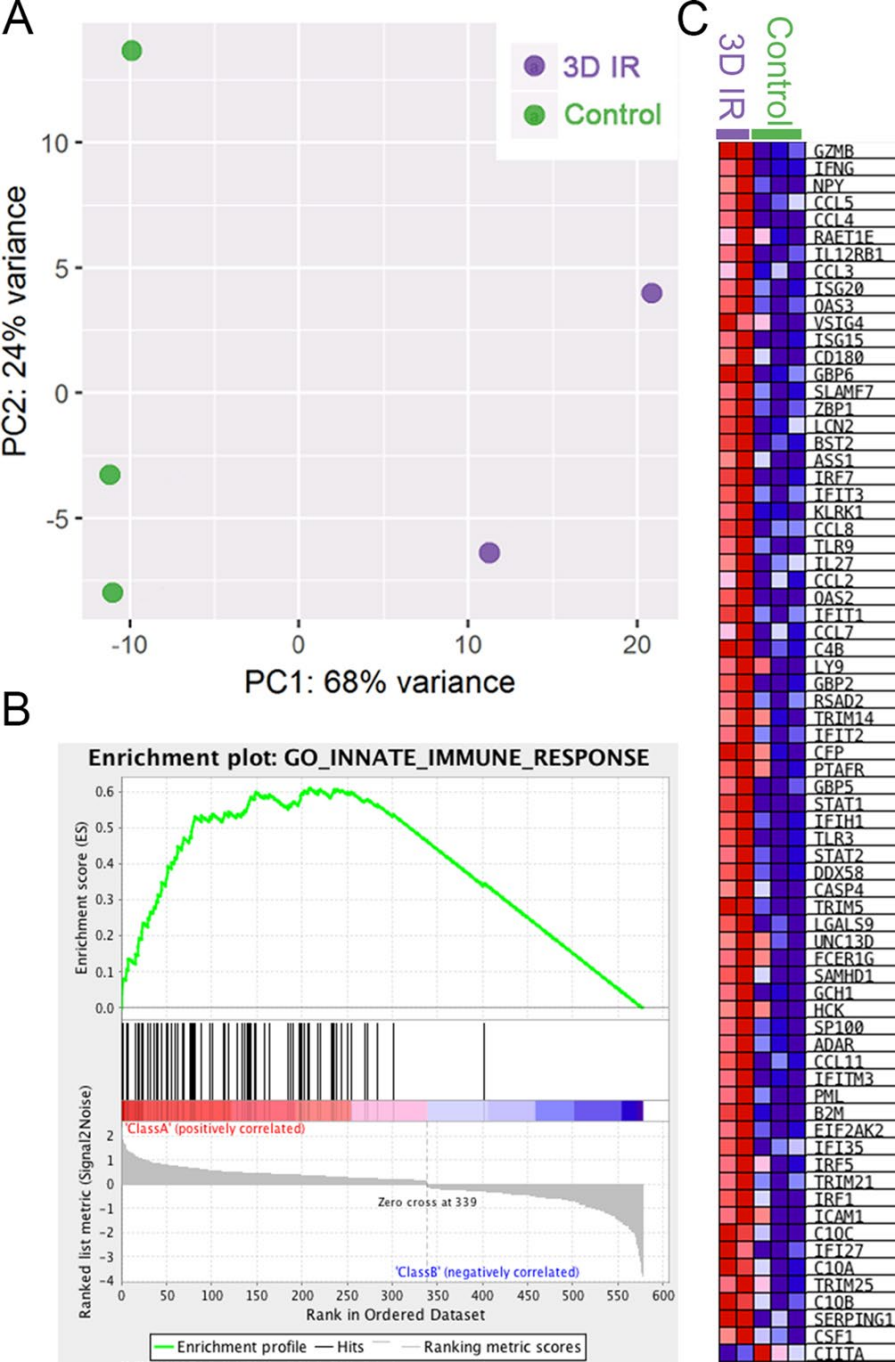
RNA-sequencing results comparing gene expression in control (non-irradiated mdx nude TA) compared to TAs treated with 18 Gy 3 days previously (3DIR). (**A**) PCA analysis showing good separation of control and 3DIR samples across principal component 1, accounting for 68% of the variance. (**B**) Top GSEA enrichment plot showing a strong enrichment of the activation of the innate immune response (GO:0045087) in 3DIR samples (normalised enrichment score = 2.907; nominal *p* < 0.000; FDR < 0.000). (**C**) Heatmap showing differentially expressed genes (fold change ≥ 2; *p* < 0.05) that match the innate immune response dataset.

PCA analysis of RNA-Sequencing data showed good separation of irradiated versus non-irradiated control samples, with samples separating across PC1, accounting for 68% of the variance (Fig. 1A). GSEA analysis comparing differentially expressed genes to gene ontology genesets shows that most of the significantly enriched genesets match an innate immune response. The top positive enrichement was GO:0045087, GO_Innate_Immune_Response (normalised enrichement score = 2.91, false discovery rate < 0.000, Fig. 1B), suggesting an activation of the innate immune response in muscles that had been irradiated 3 days previously. 71 out of 579 differentially expressed genes (fold change ≥  ± 2; adjusted *p* value < 0.05) match this geneset (Fig. 1C). Supplementary Table 1 shows all genesets enriched with an FDR value < 0.05. The vast majority of positively correlated genesets in 3 day irradiated muscles match the activation of an innate immune response and the establishment of an inflammatory response.

### Radiation does not increase cell death in *mdx*^*nu/nu*^ or C5-/Rag2-/gamma chain mouse muscles

**Figure 2:**
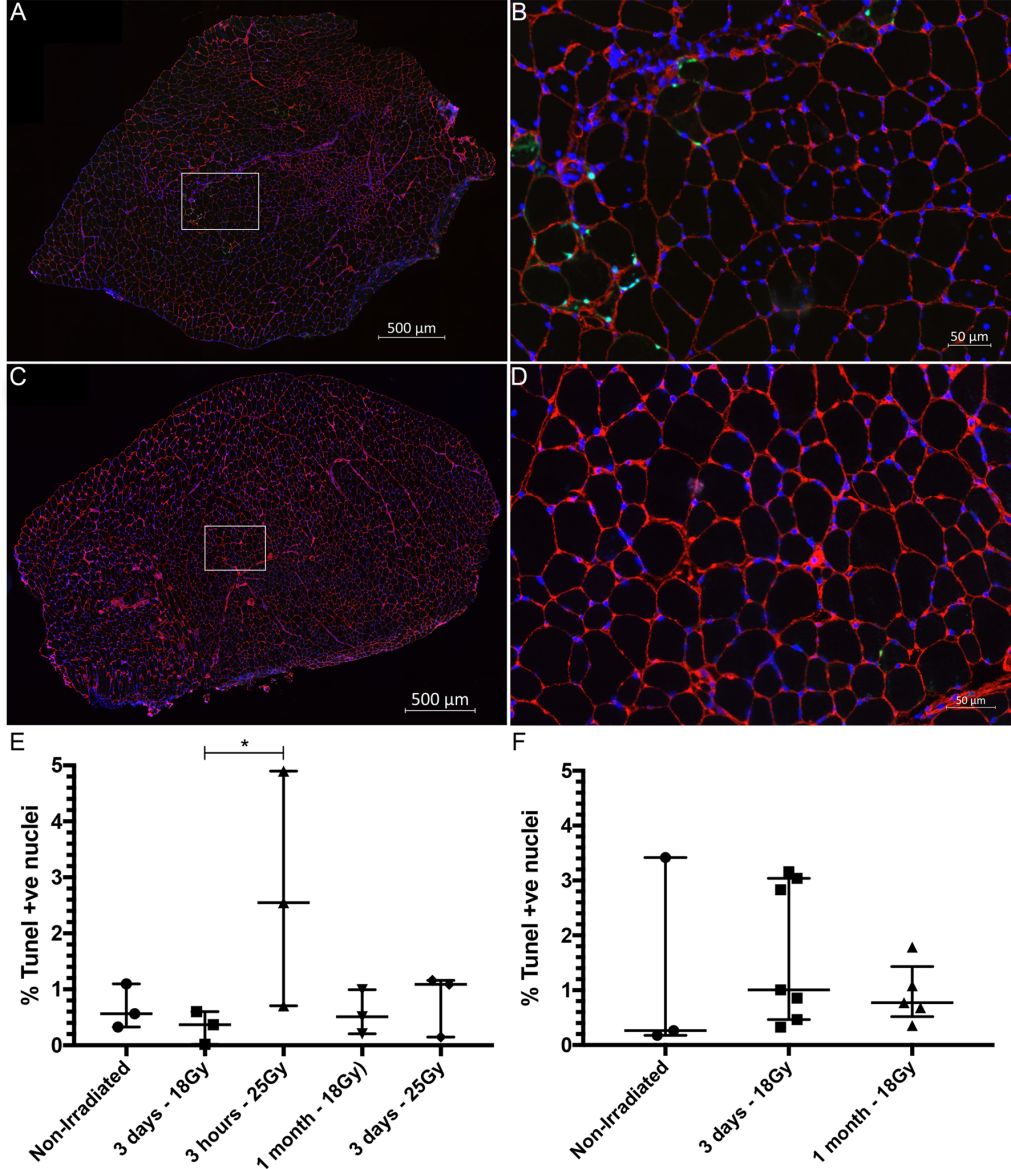
To test the hypothesis that DAMPS were responsible for the activation of an innate immune system mediate inflammatory response, the number of dying cells in the tissue were quantified using a TUNEL assay. (**A**) Section of a 3 day 18 Gy irradiated mdx nude TA stained for laminin (red) DAPI (blue) and TUNEL (green). (**B**) Augmented region (white rectangle in panel **A**) of 3 day 18 Gy irradiated mdx nude TA. (**C**) Section of a 3 day 18 Gy irradiated *C5*^−*/*−^*/Rag2*^−*/*−^*/Gamma Chain*^−*/*−^ TA stained for laminin (red) DAPI (blue) and TUNEL (green). (**D**) Augmented region (white rectangle in panel **A**) of 3 day 18 Gy irradiated *C5*^−*/*−^*/Rag2*^−*/*−^*/Gamma Chain*^−*/*−^ TA. (**E**) Quantification of the percentage of TUNEL positive nuclei in mdx nude TAs at different time points after irradiation including non-irradiated control (median = 5,650%, IQR = 1.099–0.3272, n = 3); 3 days–18 Gy (median = 0.3669%, IQR = 0.6033–0.01753, n = 3); 3 h 25 Gy (median = 2.549%, IQR = 4.898–0.7042, n = 3); 1 month–18 Gy (median = 0.5109%, IQR = 0.9917–0.2075, n = 3); and 3 days–25 Gy (median = 1.091; IQR = 1.160–0.1474, n = 3). (**F**) Quantification of TUNEL positive nuclei in *C5*^*-/*−^*/Rag2*^−*/*−^*/Gamma Chain*^−*/*−^ TAs in non-irradiated muscles (median = 0.2625, IQR = 3.419–0.1759, n = 3), 3 days after 18 Gy (median = 1.006, IQR = 3.04–0.4657, n = 7), and 1 month after 18 Gy (median = 0.7727, IQR = 1.433–0.5190, n = 5). *p < 0.05.

Activation of an innate immune response occurs via Toll-like receptor (TLR) activation, most likely initiated by damage-associated molecular pattern (DAMPS) ligands which are released from damaged or dying cells. To test the hypothesis that dying cells within irradiated host muscle might be augmenting donor satellite cell engraftment, we first assessed the amount of cell death caused by irradiation. Hindlimbs of *mdx*^*nu/nu*^ mice were irradiated, and cell death was quantified by TUNEL staining of transverse sections of TA muscles. Because the irradiation effect is both dose and time-dependent, we compared the percentage of TUNEL+ nuclei in transplantation permissive conditions (3 days after 18 Gy irradiation (n = 3 muscles) and 3 h after 25 Gy irradiation (n = 3 muscles) of *mdx*^*nu/nu*^ mouse muscles), versus non-permissive conditions (1 month after 18 Gy irradiation (n = 3 muscles) and 3 days after 25 Gy irradiation (n = 3 muscles)^3^. Controls were non-irradiated *mdx*^*nu/nu*^ TA muscles (n = 3) and non-pathological (but immunodeficient) *C5*^−*/*−^*/Rag2*^−*/*−^*/Gamma Chain*^−*/*−^ TA muscles that were either non-irradiated (n = 3 muscles), or 3 days (n = 7 muscles) or 1 month after 18 Gy irradiation (n = 5 muscles) (Fig. 2).

TUNEL+ nuclei were all outside the basal lamina (Fig. 2B, D), suggesting that the cells that are dying at these timepoints are neither satellite cells nor myofibres. In *mdx*^*nu/nu*^ mouse muscles, there were no significant differences between the percentage of TUNEL+ nuclei in non-irradiated muscles, muscles that had been given 18 Gy either 3 days or 1 month previously, or muscles that had been given 25 Gy 3 days previously (Fig. 2E). At 3 h after 25 Gy, *mdx*^*nu/nu*^ muscles contained significantly more TUNEL+ nuclei than muscles that had been given 18 Gy 3 days previously (*p* < 0.05) (Fig. 2E), although both of these treatments significantly augmented donor mouse satellite cell engraftment^3^. In *C5*^−*/*−^*/Rag2*^−*/*−^*/Gamma Chain*^−*/*−^ mice, there was no significant difference in the percentage of TUNEL+ cells in TA muscles that were non-irradiated, or that had been given 18 Gy either 3 days or 1 month previously.

Our findings suggest that increased donor satellite cell engraftment is not linked to any increase in numbers of dying cells within the host muscle.

### Donor satellite cell engraftment is not greatly enhanced when cells are transplanted into non-pathological muscles of host mice that are deficient in NK cells

To determine if donor satellite cell engraftment is enhanced within non-pathological irradiated host muscle, satellite cells from *ßactinGFP* donors were grafted into the TAs of non-irradiated and 3 day post-18 Gy irradiated *C5-/γ chain-/Rag2-* mouse hindlimbs. As positive controls, *ßactinGFP* satellite cells were grafted into the TAs of 18 Gy pre-irradiated *mdx*^*nu/nu*^ host muscles. We used *ßactinGFP* donors, as *C5-/γ chain-/Rag2-* mice are not dystrophin-deficient, so dystrophin cannot be used as a marker of muscle of donor origin in these host mice.

**Figure 3:**
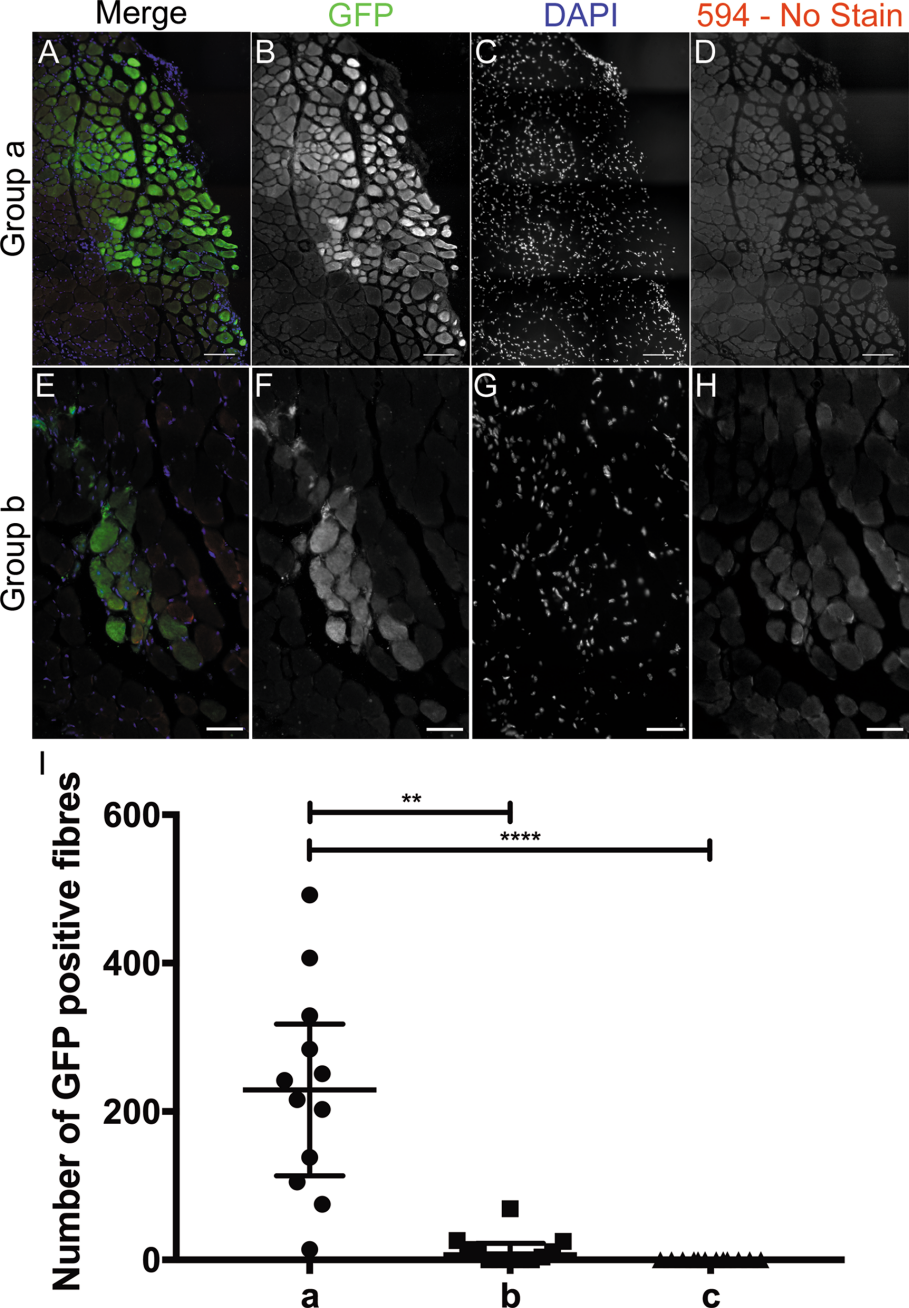
Representative images showing GFP positive fibres of donor origin in pre-irradiated mdx^nu/nu^ mice (Group a, figures **A**–**D**), and C5-/Rag2-/gamma chain- mice (Group b, figures **E**–**H**) grafted with *ßactinGFP* satellite cells 3 days after 18 Gy irradiation, and collected 1 month after transplantation; (**D**) and (**H)** (594) are shown as a reference for background autofluorescence in immersion fixed muscles. (**I**) Quantification of fibres of donor origin in 18 Gy pre-irradiated *mdx*^*nu/nu*^ mice (a, median = 229.0, IQR = 317.8–113.3, n = 12), 18 Gy pre-irradiated C5-/Rag2-/gamma chain- mice (b, median = 7, IQR = 22.25–0, n = 12), and non-irradiated C5-/Rag2-/gamma chain- mice (c, median = 0.00, IQR = 0–0, n = 12), showing a significantly higher amount of muscle of donor origin in mdx^nu/nu^ mice; (**A**–**D**) scale bars = 100 µm; (**E**–**H**) scale bars = 50 µm **p < 0.01; ***p < 0.0001.

In irradiated *mdx*^*nu/nu*^ muscles, donor satellite cells produced large amounts of muscle of donor (GFP+) origin (Fig. 3A, B), with a median of 229 (interquartile range (IQR): 317.8–113.3; n = 12) fibres of donor origin (Fig. 3I). In contrast, cells grafted into pre-irradiated *C5-/γ chain-/Rag2-* muscles gave rise to few fibres of donor origin (a median of 7 (IQR: 22.25-0; n = 12)) (Fig. 3E–F), significantly lower than those grafted into *mdx*^*nu/nu*^ mice (*p* = 0.0019). In non-irradiated *C5-/γ chain-/Rag2-* muscles, there were no fibres of donor origin (median: 0; IQR: 0–0; n = 12), significantly lower (*p* < 0.0001) than in pre-irradiated *mdx*^*nu/nu*^ host muscles (Fig. 3I). Although the amount of donor-derived muscle in pre-irradiated *C5-/γ chain-/Rag2-* muscles is negligible compared to pre-irradiated *mdx*^*nu/nu*^ muscles, it is significantly higher than in non-irradiated *C5-/γ chain-/Rag2-* muscles (*p* = 0.0046) (Fig. 3I). Even within a non-pathological muscle environment and in a host that has a deficient innate immune response, host muscle irradiation has a positive, but very small, effect on donor satellite cell engraftment.

We next wondered if the potent radiation effect within *mdx*^*nu/nu*^ muscles was due to cells that survived irradiation within the pathological muscle.

### Cells derived from mdx^nu/nu^ mouse muscles that had been irradiated with 18 Gy do not significantly enhance donor satellite cell engraftment within non-irradiated mdx^nu/nu^ host mouse muscles

**Figure 4:**
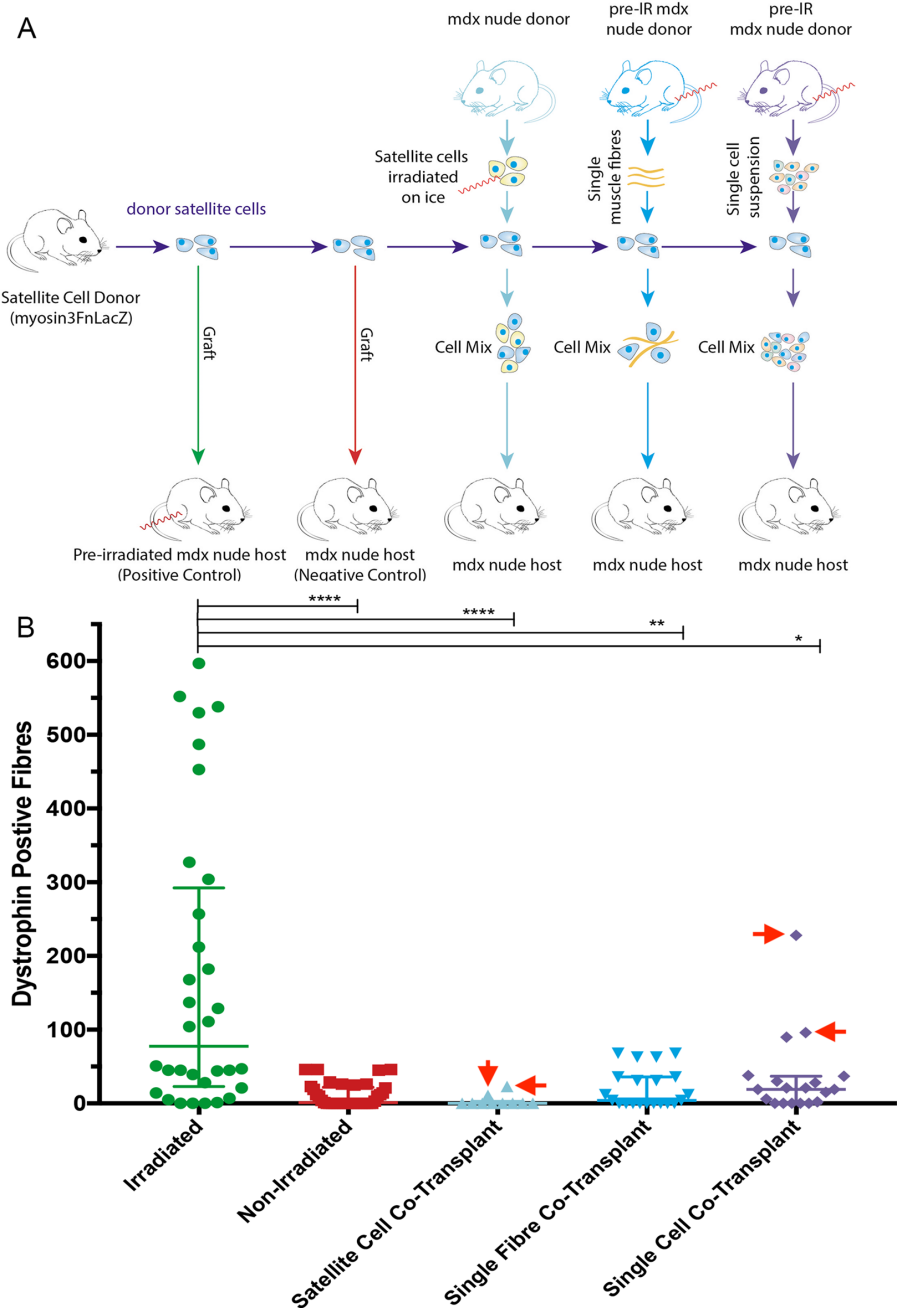
(**A**) Schematic representation of all co-transplantation experiments, including positive and negative control grafts, along with satellite cell, single fibre, and single cell suspension co-transplants. All irradiated muscles and cells received a dose of 18 Gy. (**B**) Quantification of the number of fibres of donor origin in all co-transplantation experiments. Donor satellite cells grafted into pre- irradiated host muscles (positive controls) performed significantly better than the single cell co-transplants (*p* < 0.05); single fibre co-transplants (*p* < 0.01); satellite cell co-transplants (*p* < 0.0001); and non-irradiated controls (*p* < 0.0001). There were no statistically significant differences between any of the co-transplants, or between the co-transplants and the negative controls. Data analysed by a ROUT test to identify outliers (Q = 0.1%) leading to the removal of highlighted points (red arrows), followed by a Kruskal–Wallis test *p < 0.1; **p < 0.01; ****p < 0.0001.

To determine what cells within the irradiated host muscle might be enhancing donor satellite cell engraftment, we co-transplanted donor satellite cells with different cell fractions isolated from irradiated *mdx*^*nu/nu*^ mouse muscles: satellite cells, myofibres (bearing their attendant satellite cells) or a mixture of all mononuclear cells. As we know that 50% of *mdx*^*nu/nu*^ satellite cells are already dead 3 days after irradiation^3^, we did not extract satellite cells from irradiated muscle, but instead prepared satellite cells from non-irradiated *mdx*^*nu/nu*^ EDL muscles and then irradiated them with 18 Gy. 400 donor *3F-nLacZ-2E* satellite cells were co-transplanted with either 400 *mdx*^*nu/nu*^ satellite cells that had been irradiated with 18 Gy (into n = 10 non-irradiated host muscles), or 2 myofibres (into n = 11 non-irradiated host muscles), or 1.5 × 10^4^ mixed cells (myofibres and mixed cells both being derived from an *mdx*^*nu/nu*^ TA muscle that had been irradiated with 18 Gy 3 days previously (into n = 19 non-irradiated host muscles). As controls, 400 donor *3F-nLacZ-2E* satellite cells were grafted alone into *mdx*^*nu/nu*^ host muscles that had either been irradiated with 18 Gy 3 days previously (positive control; n = 32), or were non-irradiated (negative control; n = 37) (Fig. 4A).

**Figure 5:**
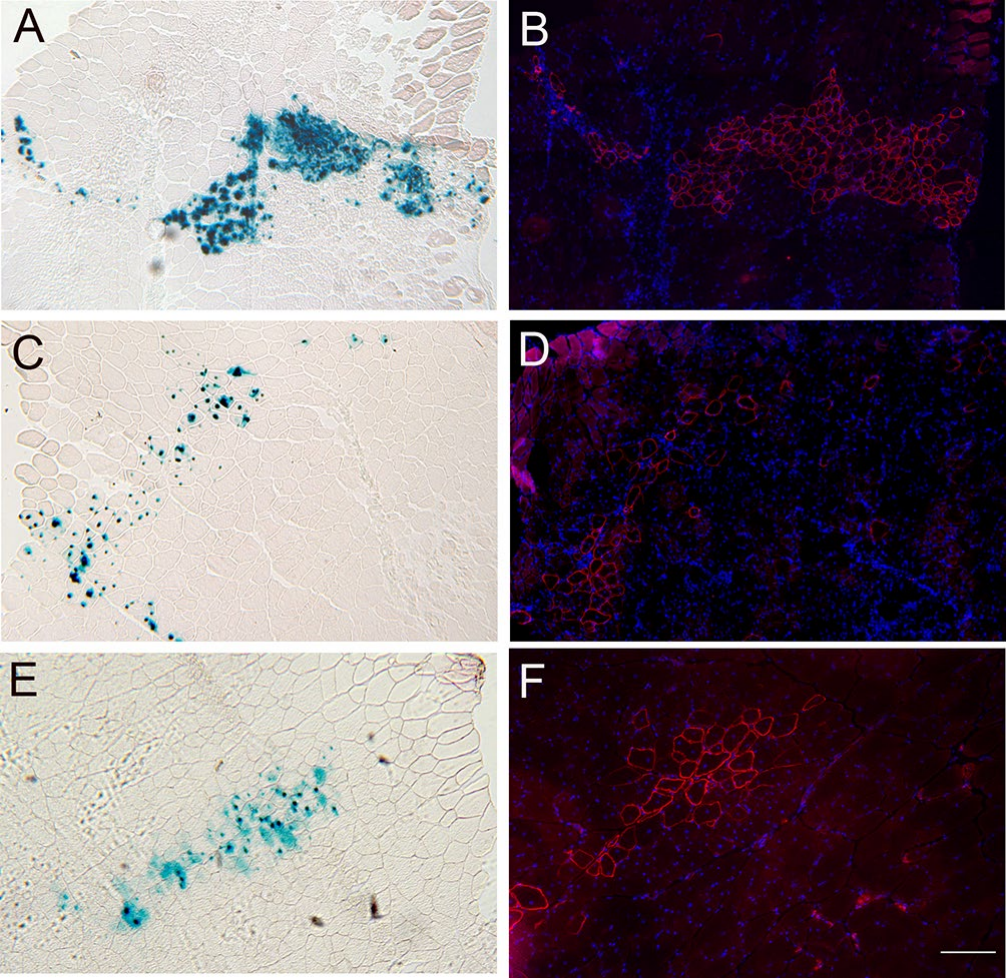
Representative images of X-gal and Dystrophin staining in serial transverse cryosections of mdx^nu/nu^ TA muscles grafted with donor satellite cells (**A** and **B** respectively); pre-irradiated single cell co-transplant (**C**, **D**); pre-irradiated single fibre co-transplant (**E**, **F**). In (**A**) and (**B**), the host muscles had been irradiated 3 days previously with 18 Gy and in (**C**–**F**), the host mdx^nu/nu^ TA muscles were non-irradiated. All scale bars = 100 µm.

As expected^3^, we found significantly more muscle of donor origin in *mdx*^*nu/nu*^ host muscles that had been irradiated with 18 Gy (Fig. 4B) compared to non-irradiated host muscles (Fig. 5). Co-transplanting donor satellite cells with either satellite cells (Fig. 4B), or myofibres (Figs. 4B, 5), or a mixture of cells derived from irradiated *mdx*^*nu/nu*^ muscle (Figs. 4B, 5) did not significantly augment the amount of muscle of donor origin within non-irradiated host muscles (Fig. 4I). But there were some outliers (ROUT test Q < 0.1%) in the mixed cell co-transplant group that performed relatively well-one grafted muscle had 238 fibres of donor origin, and another two muscles had 90 and 96 fibres of donor origin respectively (Figs. 4I, 5), suggesting that some of the cells derived from irradiated muscle may be producing factors that enhance satellite cell engraftment.

## Discussion

Our data suggest that activation of the innate immune system is necessary for efficient engraftment of transplanted satellite cells. Genes involved in the innate immune response are upregulated 3 days after *mdxnu/nu* mouse muscles were irradiated with 18 Gy, when they are permissive to donor satellite cell engrafment^3^. Evidence to support the involvement of the innate immune system in enhancing donor satellite cell function is provided by experiments in which we compared donor satellite engraftment in *C5*^−^/*γ chain*^−^*/Rag2*^−^and *mdx*^*nu/nu*^ host mice. We found significantly less muscle of donor origin derived from the same preparation of cells grafted into irradiated *C5*^−^*/γ chain*^−^*/Rag2*^−^compared to *mdx*^*nu/nu*^ muscles (Fig. 3). This may be because, in contrast to *nu/nu* mice, *C5*^−^/*γ chain*^−^/*Rag2*^−^ mice lack NK cells and have a defective innate immune system^10,11^. The fact that there is a defective innate immune response in *C5*^−^*/γ chain*^−^*/Rag2-* muscles might, at least partially, explain why donor cells engraft less well within irradiated *C5*^−^*/γ chain*^−^*/Rag2-* than *mdx*^*nu/nu*^ muscles.

The release of intracellular molecules (damage-associated molecular patterns (DAMPS)) into the extracellular space by injured tissues, elicits an inflammatory response which promotes regeneration (reviewed by^12^). Damage caused by ionising radiation has been shown to induce several Toll-like receptors (TLRs), as well as DAMPS which are ligands to the TLRs^13^. The release of debris and DAMPS from damaged or dying cells will lead to the activation of a type I interferon response within the surrounding tissue and the activation of signalling pathways such as the interferon alpha and then interferon gamma pathways, as well as TNFα via NFκB signalling from the activation of TLRs and IFNγ signalling. Pro-inflammatory cytokines (IL-1α, IL-13, TNF-α, and interferon gamma) greatly enhance the capacity of cultured primary mouse myoblasts to proliferate, and to form muscle of donor origin upon grafting in vivo^14^. DAMPS released by the cellular debris of dying, or surviving but injured, cells after irradiation, may trigger a pro-inflammatory response within the host muscle, that augments donor satellite cell engraftment. This is in keeping with findings from systems other than skeletal muscle showing that dying cells induce proliferation of neighbouring cells^9,15–19^. But there were no greater numbers of dying cells within mouse muscle at the times after the doses of irradiation that are known to promote donor cell-mediated muscle regeneration.

Satellite cell function is affected by many other cells within skeletal muscle. Signals responsible for the modulation of muscle growth or hypertrophy are released from myofibres^20–22^. In addition to differentiated muscle fibres, skeletal muscle contains stromal cells, including macrophages, fibroblasts and fiboadipogenic cells (FAPS). Stromal cells contribute to the extracellular matrix and also secrete growth factors that influence muscle regeneration (reviewed^23,24^). FAPS, that have both fibroblastic and adipogenic potential, are a source of trophic factors and play a role in muscle regeneration^25,26^. Macrophages have an effect on satellite cell function, with M1 pro-inflammatory macrophages promoting satellite cell or myoblast proliferation, enhancing their migration and delaying their differentiation^27,28^. Satellite cells and endothelial cells^29^ and satellite cells and fibroblasts^30^ interact reciprocally within regenerating skeletal muscle.

Satellite cells, particularly those in *mdx* muscles, are mostly killed or incapacitated by 18 Gy radiation, although some are radiation-resistant and can still contribute to muscle regeneration^3,31^. Although myofibres are not killed by 18 Gy of radiation^32^, there is evidence that irradiated myofibres are more leaky^33^, but it is not known what specific factors are released in response to radiation. Irradiation is known to affect macrophages, endothelial cells and fibroblasts, but its effect on FAPS is not known. Irradiation can modulate primary human macrophages towards a pro-inflammatory phenotype in vitro, leading to the activation of NFκB^34^. Whole body X-ray irradiation has also been shown to stimulate the secretion of pro-inflammatory cytokines from mouse peritoneal macrophages, increasing the secretion of TNFα, IL-1ß, IL-12 and IL-18, paralleled by the activation of NFκB as well as the up-regulated expression of CD14, TLR4-MD2 and MyD88 in a dose-dependent manner^35^. Irradiated murine embryonic fibroblasts have been shown to trigger the proliferation of murine epidermal keratinocyte progenitor cells (in vivo and in vitro), neural stem cells (in vitro), and mesenchymal stem cells (in vitro) in a caspase 3 and 7 dependent manner^9^.

We therefore investigated whether cells that had survived within irradiated *mdx*^*nu/nu*^ muscle could significantly increase the amount of muscle formed from donor satellite cells. But neither irradiated satellite cells, nor myofibres derived from irradiated *mdx*^*nu/nu*^ muscles, enhanced donor satellite cell engraftment within non-irradiated *mdx*^*nu/nu*^ muscles. This is unlikely to be an issue of timing of the irradiation, as donor satellite cells transplanted into *mdx*^*nu/nu*^ muscles that had been irradiated 3 h or 3 days previously promoted similar levels of donor satellite cell engraftment^3^. Likewise, the mixed cell population derived from irradiated *mdx*^*nu/nu*^ muscles did not elicit a statistically significant increase in donor satellite cell engraftment. But there is a hint that some cell type, which might have been a minor component of the cell mixture, is effective, as three grafted muscles had 238, 90 and 96 fibres of donor origin respectively (Fig. 4B), greatly exceeding the amount of donor-derived muscle found in non-irradiated *mdx*^*nu/nu*^ host muscles transplanted with donor satellite cells alone (Figs. 4B, 5)^3^. It is possible that we either did not inject enough cells, or that cells from irradiated muscles may have survived in insufficient numbers within each transplanted muscle to have a substantial, reproducible effect upon the co-transplanted donor satellite cells.

This mixed population would contain macrophages, endothelial cells, fibroblasts, and FAPS as well as satellite cells, myoblasts, pericytes and dendritic cells^36,37^ (reviewed^38^). It would be most interesting to know which of these cells promoted engraftment, but it would be impossible to isolate sufficient viable cells of each cell type from irradiated muscle to be able to perform co-transplant experiments to determine this.

We found significantly less muscle of donor origin derived from the same preparation of cells grafted into irradiated *C5*^−^*/γ chain*^−^/*Rag2*^−^compared to *mdx*^*nu/nu*^ muscles (Fig. 3), which might be explained by the lack of an innate immune system in *C5*^−^*/γ chain*^−^*/Rag2*^−^mice. We suspect that it may also have been because the relevant cell type was either not present at all, or not in sufficient numbers, in non-pathological muscle. There would certainly be far fewer macrophages within non-pathological muscles. Also, FAPS, fibroblasts and satellite cells would be activated and proliferating in mdx muscles and consequently be more sensitive to radiation and thus more likely to be damaged or dying, than their more quiescent counterparts in normal mouse muscles. But irradiation of *C5*^−^*/γ chain*^−^*/Rag2*^−^ muscle does have a small, but statistically significant, positive effect on donor satellite cell (Fig. 3I) and a greater effect on conditionally-immortal myoblast engraftment^2^, suggesting that the relevant cells must be present in non-pathological muscles.

Our findings are of interest from a biological, as well as a translational, point of view. They suggest that it might be possible to enhance donor stem cell engraftment within skeletal muscle by adding appropriate factor(s) at the time of cell transplantation. From a practical aspect, when considering a mouse model for skeletal muscle intramuscular stem cell transplantation, one must take into account the pathological environment, as well as the immunological status of the host mouse and the species of donor cell^3,39^, as immune signalling proteins may be species-specific^40^. And for intra-muscular stem cell therapy to be effective clinically, patients might have to be capable of mounting a timely innate immune response, in order for transplanted stem cells to efficiently engraft skeletal muscle.

A major limitation of our work is that we neither identify the cells responsible, nor the mechanism(s) by which they are eliciting the radiation-mediated enhancement of donor satellite cell engraftment. Our findings however set the groundwork for future studies. The effector cells are unlikely to be satellite cells or myofibres, but our evidence suggests that they are interstitial cells (e.g. macrophages, fibroblasts, endothelial cells or FAPS). Although macrophages are a good candidate, as they have already been shown to enhance the muscle regenerative capacity of myoblasts transplanted into mice^28,41^ and are one of the most radiation-resistant cell types^34^, other cells, or a mixture of cell types, might be responsible. And the mechanism(s) by which they are mediating enhanced donor satellite cell engraftment remains to be elucidated. However, our findings suggest that the innate immune response may be an important factor in enhancing satellite cell function. In support of this hypothesis, it has recently been shown that the activation of the innate immune system by freeze/thaw killed cells, or a chemical activator, was able to elicit a localised inflammatory response which aided cardiac regeneration after ischemia reperfusion injury^42^. We speculate that the activation of the innate immune response after sterile radiation injury of the host muscle triggers a localised inflammatory response which is able to enhance donor satellite cell expansion within the host muscle.

## Methods

### Host and donor mice

Mice were bred and experimental procedures carried out in the Western Laboratories, Biological Services Unit, University College London Great Ormond Street Institute of Child Health, in accordance with the Animals (Scientific Procedures) Act 1986. All experiments were approved by the University College London Animal Welfare Ethical Review Body and carried out under Home Office Licence. Host mice were either *mdx*^*nu/nu*^^43^, or *C5-/γ chain-/Rag2-*^2^. The mean weight of *mdx*^*nu/nu*^ host mice was 20 g and the mean weight of *C5-/γ chain-/Rag2-* mice was 25 g. We used both male and female mice, as we have previously shown that sex of the host mouse does not affect donor satellite cell engraftment^3,44,45^. Donor satellite cells were prepared from either *3F-nLacZ-E*^3,46^*, or ßactinGFP* mice^47^.

### Irradiation of mouse muscles

*Mdx*^*nu/nu*^*,* or *C5-/γ chain-/Rag2-* mice were anaesthetised and hindlimbs irradiated with either 18 or 25 Gy, as described previously^3^. Controls were anaesthetised, but not irradiated. Mice were either used as hosts for cell transplant experiments, or their muscles were removed for either cell preparation, or analysis.

### RNA sequencing

TA muscles from 3 to 4 week old male *mdx*^*nu/nu*^ mice whose hindlimbs had been irradiated with 18 Gy 3 days previously (2 mice) and age and sex-matched non irradiated controls (n = 3) were placed in sterile RNAse free DNAse free Eppendorf tubes and immediately snap frozen in liquid N_2_ and kept at − 80 °C. RNA extractions were performed using the *mir*Vana™ Isolation Kit (Ambion AM1560) according to the manufacturer’s instructions. The quality and concentration of the extracted RNA was first assessed using a NanoDrop 1,000 spectrophotometer (Thermo-Scientific) ensuring the A_260_/A_280_ value was approximately 2.0. The integrity of the extracted RNA was determined using the 2,200 TapeStation system (Aligent G2964AA), RNA ScreenTape (Aligent 5,067–5,576) and the Aligent TapeStation software. Sequencing was performed by the Genomics Services Laboratory at Nationwide Children’s Hospital, by Dr Peter White’s group. Sequencing was performed at 50 million 150 bp paired end reads to maximise the accurate alignment of RNA reads. They also analysed the raw data. Their parameters were set so each sample was aligned to the GRCm38.p4 assembly of the mouse reference from NCBI, using version 2.5.1b of the RNA-Seq aligner STAR^48^, and features were identified from the GFF file included with the assembly genome from NCBI. Coverage counts were calculated using HTSeq^49^ and differentially expressed features were calculated using DESeq2^50^. A spreadsheet were returned that contained features with an absolute fold change ≥ 2 and an adjusted FDR *p* value ≤ 0.10, and also quality control PCA, volcano, MAV plots, alignment counts and read lengths. From these data, we present Principal Component Analysis (PCA) plots. Gene Set Enrichment Analysis (GSEA) was also performed^51,52^, using the molecular signatures database, using the gene ontology gene set collection, as a reference, on the full set of differentially expressed genes returned from RNA sequencing. Positively and negatively enriched genesets with an FDR value < 0.05 are shown in Supplementary Table 1. A heatmap was constructed of differentially expressed genes (fold change ≥ 2; *p* < 0.05) that match the innate immune response dataset.

### Cell preparation

Satellite cells were prepared from isolated myofibres derived from EDL muscles of donor *3F-nLacZ-E, ßactinGFP* and *mdx*^*nu/nu*^ mice^3^. Satellite cells from *mdx*^*nu/nu*^ mice were suspended in plating medium^53^ on ice and irradiated with 18 Gy. Cells were kept on ice for approximately 3 h before grafting.

Myofibres were prepared from EDL muscles^22,54^ of *mdx*^*nu/nu*^ mice, whose hindlimbs had been irradiated with 18 Gy 3 days previously. Myofibres were placed in a horse serum-coated Petri dish containing plating medium^22^ and kept at 37 °C for approximately 1–2 h and then at room temperature for approximately 1 h prior to grafting.

The cell mixture was prepared from lower hindlimb muscles of *mdx*^*nu/nu*^ mice, that had been irradiated with 18 Gy 3 days previously. Dissected muscles were washed in phosphate buffered saline (PBS), minced finely and disaggregated in collagenase II (500 u/ml, (Sigma C0130), USA in 2 ml DMEM) at 37 °C for 30 min. After digestion, the muscle fragments were crushed using the flat end of a sterile 5 ml syringe plunger to create a ‘sludge’. 5 ml of cold PBS containing 10% foetal bovine serum (FBS, GIBCO 10270-106, Thermofisher, USA) was then added to the muscle preparation and transferred to a 50 ml falcon tube. Another 5 ml of PBS/10%FBS was used to rinse the dish and added to the falcon tube. The suspension was then shaken vigorously and the volume made up to 50 ml with PBS/10% FBS. The suspension was then centrifuged at room temperature at 600* g* for 5 min. The supernatant was then discarded, the pellet resuspended in PBS/10% FBS and centrifuged again at 600 g. The pellet was then resuspended in 1 ml of a collagenase D/Dispase II solution (collagenase D (Roche Diagnostics UK ref: 11088866001) 1.5 u/ml and Dispase II (Roche 14549000) 2.4 u/ml). The suspension was incubated for 1 h at 37 °C, and triturated with a 1 ml Gilson pipette every 15 min. After 1 h, the suspension was placed on ice. 50 ml of PBS/10%FBS were added, and the suspension triturated by repeated pipetting. The suspension was then filtered through a 40 µm cell strainer and centrifuged at 1,500 rpm for 5 min in a ALC PK130 centrifuge. The supernatant was then removed to a new falcon tube and centrifuged at 2,500 rpm for 5 min^55^. The pellet was resuspended in 1 ml of plating medium (in preparation for co-transplant experiments) and kept on ice. The number of live cells/ml was counted by staining a 2 µl aliquot of the cell suspension with 2 µl of 0.4% trypan blue (Invitrogen T10282, Thermofisher, UK), placed on counting slides (Bio-Rad, UK 145-0011) on a TC20 automated cell counter (Bio-Rad, UK). Two counts were made for each cell suspension and the average was used to adjust the live cell concentration to 7.5 × 10^3^ cells/µl for grafting (2 µl per graft, equivalent to 1.5 × 10^4^ cells).

### Cell transplantation

Mice were anaesthetised and cells transplanted as described previously^22^. For myofibre co-transplants, two muscle fibres were collected using a glass micro-pipette and injected into the muscle, followed by 400 *3FTGnLacZ* satellite cells. For co-transplants of donor satellite cells with the cell mixture, 1.5 × 10^4^ cells from the mixed population were transplanted with 400 donor satellite cells, in a total volume of 5 µl.

For co-transplants of donor satellite cells with irradiated satellite cells, 400 cells of each cell type were injected in a total volume of 4 µl.

### Muscle processing and staining

Grafted muscles were removed 28 days after cell transplantation, for analysis. Muscles that had been transplanted with *3FTGnLacZ* satellite cells were bisected transversely and mounted in 6% gum tragacanth on cork disks and immediately frozen in liquid N_2_ cooled isopentane (VWR UK 103616 V). Once frozen, they were placed into liquid nitrogen, and then kept at − 80 °C. Serial 10 µm transverse cryosections were cut and either fixed and stained with X-gal or immunostained with an anti-dystrophin antibody (custom made P7 antibody^56^ and counterstained with DAPI (10 µg/ml)^44^.

Muscles that had been grafted with *ßactinGFP* donor satellite cells were fixed in cold 4% paraformaldehyde (PFA) in PBS overnight at 4 °C. After fixation, the tissues were dehydrated in 30% (w/v) sucrose (Fisher, UK S/860/053) in PBS overnight at 4 °C. Finally, the muscles were bisected transversely and mounted in 6% gum tragacanth on cork disks and immediately frozen in liquid N_2_ cooled isopentane, then stored at − 80 °C. Serial 10 µm transverse cryosections were immunostained with a rabbit anti GFP antibody for 2 h at room temperature (RT) (Invitrogen A6455, Thermofisher, UK 1:1,000) followed by goat anti-rabbit Alexa Fluor 488 for 1 h at RT (Invitrogen A11034, Thermofisher, UK 1:1,000) and counterstained with DAPI (10 µg/ml).

For TUNEL staining, irradiated and control muscles were removed 3 h, 3 days or one month after irradiation. TUNEL+ nuclei were detected using the “ApopTag® Fluorescein In Situ Apoptosis Detection Kit” (Merck Millipore, USA S7110), which is based on detecting DNA fragmentation that occurs during apoptosis. The kit was used according to the manufacturer’s instructions, using 7 µm serial sections at least 100 µm apart. After dioxigenin staining, the sections were stained with a rabbit pan-laminin antibody (L9393, Sigma Aldrich,USA 1:500), followed by goat anti-Rabbit AlexaFluor 594 (A11037, Invitrogen,Thermofisher, USA 1:1,000), prior to mounting in Hydromount mounting media containing DAPI (10 µg/ml). The stained samples were stored at − 20 °C until required.

### Image capture and quantification

Bright-field microscopy and fluorescence microscopy was performed using a Leica DM4000B microscope and captured using MetaMorph® software (Molecular Devices Inc, USA). For scanning full sections, the scan slide function was used. The images were then stitched together to form a composite image of the entire transverse section using MetaMorph® software.

Fibres of donor origin in host muscles grafted with *3FTGnLacZ* satellite cells were quantified by first identifying areas of muscle of donor origin (x-gal positive areas)^44,46^. Serial sections were then stained for dystrophin^44^ and the section with the largest amount of dystrophin positive fibres was imaged with the “scan slide” function in Metamorph. Using the count tool on Adobe Photoshop CC (2015), dystrophin positive fibres that coincided with X-gal positive areas on serial sections were counted. Small, isolated groups of dystrophin positive fibres, many of which did not have dystrophin around the entire myofiber circumference and which did not co-localise with X-gal positive areas, were omitted, as these are likely to be rare revertant fibres, that occur naturally in mdx mouse muscles^57^.

GFP stained muscle sections were imaged using the scan slide function. Three channels were captured: red, green and blue. Red was used to measure the average background fluorescence from PFA fixation; only GFP positive fibres exceeding background level auto fluorescence were counted using the count tool on Adobe Photoshop CC (2015).

For TUNEL quantification, stained slides were scanned with a ZEISS Axio Scan.Z1 Slide Scanner (Carl Zeiss Microscopy GmbH, Germany). The total number of DAPI+ nuclei and TUNEL+ nuclei (co-localising with DAPI) were counted in 5 sections from each muscle (spaced 100 µm or more apart), using a macro in FIJI^58^ described in the Supplementary materials, and the results presented as a percentage of the total number of nuclei. Positive controls were sections from wax embedded receding rat mammary gland (provided with the kit). Additionally, to test the efficacy of the kit on frozen tissues, sections from CD1 newborn mouse thymus were used as positive controls.

Minor modifications to brightness, contrast, and pseudo-colouring of images were performed using Fiji (NIH) or Photoshop CC 2015, all modifications were applied to the whole image. Quantification of transplanted muscles was performed by using the count tool included in Photoshop. Figures and images were arranged using either Adobe Photoshop CC (2015) or Illustrator CC (2015).

### Statistical analyses

Results from cell grafting experiments with more than 2 groups were analysed using a Kruskall–Wallis test with a Dunn’s multiple comparisons test. If only two groups were compared a Mann–Whitney test was employed. TUNEL assay results were analysed using a one-way ANOVA with a Tukey’s multiple comparisons test.

## Supplementary information

Supplementary file1

Supplementary file2
