# Supplementary material for: Irradiation dependent inflammatory response may enhance satellite cell engraftment
Source: Sci Rep. 2020 Jul 6;10:11119. doi: 10.1038/s41598-020-68098-9 (PMC7338540; doi:10.1038/s41598-020-68098-9)
Supplement: Supplementary file 1 — Supplementary file1 [file 41598_2020_68098_MOESM1_ESM.pdf]

Irradiation dependent inflammatory response may enhance satellite cell engraftment

Bruno Doreste, Silvia Torelli, Jennifer Morgan

Supplementary data

Macro for quantifying TUNEL+ cells

Macros ran on FIJI. The DAPI count was written by Dale Moulding. The TUNEL count was adapted by B.D. for counting TUNEL positive cells.

DAPI count

```
dir1 = getDirectory("Input folder"); //select an input folder
dir2 = getDirectory("Choose a folder to save to"); //select an output folder.
list = getFileList(dir1); //make a list of the filenames
setBatchMode(true); //turn on batch mode so it runs in the background
        if (isOpen("Results")) {
             selectWindow("Results");
             run("Close");
          }

// repeats the macro for every file in the folder using a for loop.
        for (i=0; i<list.length; i++) {
        showProgress(i+1, list.length);
        filename = dir1 + list[i];

        open(filename);
        Imagename = File.nameWithoutExtension;

        // set the value for the Gaussians
        GausLow = 7;
        GausHigh = 10;
        run("Brightness/Contrast...");
        run("Enhance Contrast", "saturated=0.35");
        run("Duplicate...", "title=GausLow duplicate channels=3");
```

```
	run("Duplicate...", "title=GausHigh");
	run("Gaussian Blur...", "sigma="+GausHigh);
	selectWindow("GausLow");
	run("Gaussian Blur...", "sigma="+GausLow);// set the value for the low gaussian
	imageCalculator("Subtract create", "GausLow","GausHigh");
	selectWindow("Result of GausLow");
	run("Find Maxima...", "prominence=15 output=Count");
	setResult("Image", i, Imagename);
	updateResults();
	selectWindow("Result of GausLow");
	run("Find Maxima...", "prominence=15 output=[Point Selection]");
	roiManager("Add");
	selectWindow("GausLow");
	close();
	selectWindow("GausHigh");
	close();
	number=roiManager("count");
	roiManager("select", number-1);
	roiManager("Rename", "Dapi_"+Imagename);
	run("Close All");
	}
roiManager("Save", dir2+"Nuclei-RoiSet.zip"); // saves the ROIs .
selectWindow("Results");
saveAs("Results", dir2+"Nuclei-Counts.csv");// save the summary as a .csv.
exit("Nuclei measured in "+i+" images"); // close the macro and display a window with
number of images processed.
```

TUNEL Count

```
dir1 = getDirectory("Input folder"); //select an input folder
```

```
dir2 = getDirectory("Choose a folder to save to"); //select an output folder.

list = getFileList(dir1); //make a list of the filenames

setBatchMode(true); //turn on batch mode so it runs in the background

        if (isOpen("Results")) {

            selectWindow("Results");

            run("Close");

          }


// repeats the macro for every file in the folder using a for loop.

        for (i=0; i<list.length; i++) {

        showProgress(i+1, list.length);

        filename = dir1 + list[i];


        open(filename);

        Imagename = File.nameWithoutExtension;

        imagename= File.name();


        // set the value for the Gaussians

        GausLow = 2.75;

        GausHigh = 5;

        run("Split Channels");

        selectWindow(imagename+" (green)");

        run("Duplicate...", "title=GausLow duplicate channels=3");

        run("Duplicate...", "title=GausHigh");

        run("Gaussian Blur...", "sigma="+GausHigh);

        selectWindow("GausLow");

        run("Gaussian Blur...", "sigma="+GausLow);// set the value for the low gaussian

        imageCalculator("Subtract create", "GausLow","GausHigh");
```

```
	selectWindow("Result of GausLow");
	run("Find Maxima...", "prominence=30 output=Count");
	setResult("Image", i, Imagename);
	updateResults();
	selectWindow("Result of GausLow");
	run("Find Maxima...", "prominence=30 output=[Point Selection]");
	roiManager("Add");
	selectWindow("GausLow");
	close();
	selectWindow("GausHigh");
	close();
	number=roiManager("count");
	roiManager("select", number-1);
	roiManager("Rename", "TUNEL_"+Imagename);
	run("Close All");
	}
roiManager("Save", dir2+"TUNEL-RoiSet.zip"); // saves the ROIs .
selectWindow("Results");
saveAs("Results", dir2+"TUNEL-Counts.csv");// save the summary as a .csv.
exit("TUNEL measured in "+i+" images"); // close the macro and display a window with
number of images processed.
```

Supplementary Table 1. GSEA results for enriched genesets with an FDR value below 0.05, with a large amount of gene ontology enrichments related to the activation of an innate immune and inflammatory responses.
